# Sexual risk during pregnancy and postpartum periods among HIV-infected and –uninfected South African women: Implications for primary and secondary HIV prevention interventions

**DOI:** 10.1371/journal.pone.0192982

**Published:** 2018-03-06

**Authors:** Dvora Joseph Davey, Elise Farley, Yolanda Gomba, Thomas Coates, Landon Myer

**Affiliations:** 1 Division of Epidemiology and Biostatistics, School of Public Health and Family Medicine, University of Cape Town, Cape Town, South Africa; 2 Department of Epidemiology, Fielding School of Public Health, University of California, Los Angeles, Los Angeles, California, United States of America; 3 David Geffen School of Medicine, University of California, Los Angeles, Los Angeles, California, United States of America; The Ohio State University, UNITED STATES

## Abstract

**Background:**

HIV acquisition in pregnancy and breastfeeding contributes significantly toward pediatric HIV infection. However, little is known about how sexual behavior changes during pregnancy and postpartum periods which will help develop targeted HIV prevention and transmission interventions, including pre-exposure prophylaxis (PrEP).

**Methods:**

Cross-sectional study in HIV-infected and uninfected pregnant and postpartum women in Cape Town, South Africa. Interviewers collected survey data on demographic, sexual behaviors, and alcohol use among pregnant and post-partum women. We report descriptive results of sexual behavior by trimester and postpartum period, and results of multivariable logistic regression stratified by pregnancy status.

**Results:**

We enrolled 377 pregnant and postpartum women (56% pregnant, 40% HIV-infected). During pregnancy, 98% of women reported vaginal sex (8% anal sex, 44% oral sex) vs. 35% and 88% during the periods 0–6 and 7–12 months postpartum, respectively (p<0.05). More pregnant women reported having >1 partner in the past 12-months compared to postpartum women (18% vs. 13%, respectively, p<0.05). Sex frequency varied by trimester with greatest mean sex acts occurring during first trimester and >6-months postpartum (13 mean sex acts in first trimester; 17 mean sex acts >6-months postpartum). Pregnant women had increased odds of reporting condomless sex at last sex (aOR = 2.96;95%CI = 1.84–4.78) and ever having condomless sex in past 3-months (aOR = 2.65;95%CI = 1.30–5.44) adjusting for age, HIV status, and sex frequency compared to postpartum women.

**Conclusion:**

We identified that sexual behaviors and risk behaviors were high and changing during pregnancy and postpartum periods, presenting challenges to primary and secondary HIV prevention efforts, including PrEP delivery to pregnant and breastfeeding women.

## Introduction

The risk of HIV acquisition during pregnancy remains high in South Africa despite increased access to and initiation of antiretroviral therapy (ART) [1–3], and maternal seroconversion during later pregnancy and breastfeeding contributes significantly toward pediatric HIV infection [3]. Increased susceptibility to HIV infection during pregnancy and breastfeeding may result from hormonal changes that alter genital mucosal surfaces or distribution of target cells at these surfaces [1]. However, behavioral factors also play a role in increasing HIV risk if pregnant women have higher frequency of condomless sex and sexually transmitted infections (STIs), or if they have male partners who seek other partners during pregnancy or postpartum and bring HIV back into the relationship [4–8]. There are numerous studies on the physiological changes during pregnancy and postpartum periods which increase HIV risk [9–10]. However, behavioral factors related to pregnancy and postpartum periods are less understood, yet very important, in increasing HIV acquisition and transmission risk [5, 7, 11–14]. Further, there are few studies on how risk factors vary across pregnancy and postpartum periods [6].

HIV-infected pregnant women are at risk of transmitting HIV, not only to their infants, but to their sex partners. In addition, HIV-infected pregnant women are particularly vulnerable to potential re-infection, viremia and sexually transmitted infection (STI) which may increase the risk of both vertical and horizontal HIV transmission [6–8]. A recent study demonstrated that STIs may increase the risk of mother to child HIV transmission [15]. Another study of HIV-infected South African pregnant women demonstrated that 13% had a viral load >1000 and 7% had a viral load of >10,000 copies/mL [16]. Despite this risk, there is limited understanding of sexual risk factors for HIV re-infection and onward HIV transmission in HIV-infected pregnant women.

Our study evaluated sexual behaviors across trimester and between pregnancy and postpartum periods among HIV-infected and uninfected women during pregnancy and postpartum to determine risk factors associated with increased HIV acquisition and transmission risk. Those findings have the potential to improve the implementation of counseling, risk reducing messages, and primary and secondary prevention interventions among HIV-exposed HIV-uninfected women who are pregnant, post-partum, or seek to get pregnant in South Africa and beyond.

## Methods

Between July and December 2016, we enrolled consenting pregnant or postpartum women attending primary care services to participate in a 30-minute survey. Women received information about the study during their antenatal or postnatal care visit from the study counselor, who counseled them about the risks and benefits of participating in the study. We selected a convenience sample of 2 to 5 pregnant and post-partum women per day depending on the patient flow and capacity of the counselors. Participants received reimbursement for transportation and a snack (R100, equivalent of $8 USD). All surveys were conducted in private rooms by trained study counselors in the local dialect, isiXhosa.

### Participants

We enrolled pregnant and postpartum women (child was ≤ 18 months old) who were attending primary care services at a large primary care health facility in Cape Town, South Africa. A study counselor provided information on the study and consented participants who were willing to participate in the 30-minute survey. All consenting mothers participated in an interviewer administered questionnaire which investigated participant and partner demographics, sexual behaviors during and after pregnancy, and alcohol use during pregnancy. Inclusion criteria included: (1) ≥18 years old, (2) seeking antenatal care in a health facility or post-natal mothers with infants 6–18 months old), (3) willingness to participate in the study (approximately 30-minute quantitative interview. Exclusion criteria included: (1) younger than 18 years old, (2) not pregnant, (3) not during post-natal period (with infant >18-months old), (4) not willing to participate in the study. Any ethnic or language group were eligible to participate, as long as they could provide informed consent.

### Ethics

We received institutional review board approval from University of Cape Town and University of Los Angeles, California. Interviews were held in private rooms with a closed door at the clinic to ensure that privacy is protected. All study tools were coded and anonymous. Study counselors were trained on how to consent patients, and how to minimize undue influence using non-judgmental tones and probing questions to preface each section of the survey and encourage honest responses. All study staff were monitored regularly by the researcher team

### Analysis

We describe continuous variables as means with standard deviations where normally distributed, and as medians and inter-quartile ranges (IQR) if non-normally distributed. Binary variables were described using frequencies and percentages. Logistic regression modelling was used to analyze risk factors of HIV acquisition and transmission. We created multivariable models controlling for *a priori* covariates including mother’s age, relationship status and education. Separate analyses were performed for pregnancy vs. postpartum, and HIV-infected vs. uninfected women. Data analysis was carried out using STATA/SE statistical software package version 14.0 (StataCorp., College Station, TX, USA).

- **Exposures:** maternal age, maternal and paternal HIV status, stage of pregnancy (self-reported), stage of postpartum period (0–6 months, 7–12 months), employment status, cohabitation status, number of previous pregnancies, number of live children, and alcohol use during pregnancy (using AUDIT validated questions), including quantity of alcohol use and what quantified a drink (e.g. bottle of beer, glass of wine, etc.).
- **Outcomes**: condomless sex with partner at last sex act, frequency of sex (and condomless sex) during those periods, any condomless sex during pregnancy, frequency of sex acts per month during and after pregnancy, having a partner of unknown serostatus, and multiple partners in year prior to study enrollment.

## Results

We enrolled 212 pregnant women and 165 postpartum women, of which 40% were HIV-infected (37% of pregnant women and 44% of postpartum women). Median age was 28 years (IQR = 23, 32). Overall fewer than half of women were married or cohabiting with the father of the index pregnancy/child (48% of pregnant women vs. 40% of postpartum women) and more HIV-infected pregnant women reported being married or cohabiting more than HIV-uninfected women (56% vs. 44%). More pregnant women reported not knowing the serostatus of the father of the index pregnancy/child (44% vs 32% of postpartum women) and fewer reported being serodiscordant with their father of the index pregnancy/child (7% vs. 10% respectively). HIV-uninfected women reported being in sero-concordant relationships more than HIV-infected women (58% of HIV+ pregnant women and 73% of postpartum HIV+ women). Two-thirds of women reported being unemployed, higher among postpartum vs. pregnant women (74% vs. 62% respectively, p = 0.02). Overall, 33% of women did not want to get pregnant and 7% were unsure with no difference by serostatus. Twenty-nine percent of pregnant women reported any alcohol use during pregnancy, and 27% of women reported drinking alcohol 4 or more times a month and 2% reported drinking 2 or more times a week (no different by serostatus) (Table 1).

**Table 1:** Demographic and health behaviors in pregnant and postpartum women (n = 377).

	Total Pregnant and Postpartum	Pregnant			Post Partum			p-value
		Overall (N = 212)	HIV- (N = 134)	HIV + (N = 78)	Overall (N = 165)	HIV- (N = 92)	HIV + (N = 73)	
**Socio- Demographics**								
**Age (median, IQR)**	377	28 (24, 32)	27 (23, 32)	30 (25, 33)	28 (23, 32)%	27 (23, 31)	29 (25, 32)	0.81
**Education**								
None	1	0 (0%)	0 (0%)	0 (0%)	1 (1%)	0 (0%)	1 (1%)	0.45
Below Matric	208	112 (53%)	68 (51%)	44 (57%)	96 (58%)	52 (57%)	44 (60%)	
Matric	157	94 (44%)	61 (46%)	33 (42%)	63 (38%)	36 (40%)	27 (37%)	
Degree/diploma	11	6 (3%)	5 (4%)	1 (1%)	5 (3%)	4 (4%)	1 (2%)	
**Relationship with father of child**								
No relationship	40	18 (8%)	8 (6%)	10 (13%)	22 (14%)	8 (9%)	14 (20%)	0.22
Married/cohabiting	166	101 (48%)	58 (44%)	43 (56%)	65 (40%)	39 (43%)	26 (37%)	
Casual partner/non-cohabiting	168	92 (43%)	68 (51%)	24 (31%)	76 (47%)	45 (49%)	31 (43%)	
Widowed	1	1 (1%)	0 (0%)	1 (1%)	0 (0%)	0 (0%)	0 (0%)	
**HIV status of father of child**								
Sero-concordant	200	105 (50%)	78 (58%)	27 (35%)	95 (58%)	67 (73%)	28 (39%)	0.062
Sero-discordant	30	14 (7%)	0 (0%)	14 (18%)	16 (10%)	1 (1%)	15 (21%)	
Don’t know	146	93 (44%)	56 (42%)	37 (48%)	53 (32%)	24 (26%)	29 (40%)	
**Current employment status**								
Employed	123	80 (38%)	56 (42%)	24 (31%)	43 (26%)	24 (26%)	19 (26%)	0.018
Unemployed	253	132 (62%)	78 (58%)	54 (69%)	121 (74%)	68 (74%)	53 (74%)	
**Wanted to get pregnant this time**								
Yes	121	73 (35%)	46 (35%)	27 (35%)	48 (30%)	31 (34%)	17 (24%)	0.24
No	224	119 (57%)	74 (56%)	45 (59%)	105 (65%)	57 (63%)	48 (68%)	
Unsure	27	18 (9%)	13 (10%)	5 (6%)	9 (6%)	3 (3%)	6 (8%)	
**Alcohol questions**								
**During pregnancy frequency of alcohol consumption**								
Never	267	149 (71%)	97 (72%)	52 (68%)	118 (72%)	68 (74%)	50 (69%)	0.98
≤4 times a month	102	58 (27%)	35 (27%)	23 (29%)	44 (27%)	24 (26%)	20 (27%)	
≥2 times a week	7	4 (2%)	2 (1%)	2 (3%)	3 (2%)	0 (0%)	3 (4%)	
**During pregnancy consumed alcohol 6+ times in one occasion**								
Never	299	167 (79%)	11 (29%)	6 (25%)	132 (80%)	21 (58%)	13 (42%)	0.68
≤4 times a month	71	42 (20%)	24 (62%)	18 (74%)	29 (18%)	14 (39%)	15 (49%)	
≥2 times a week	7	3 (1%)	3 (8%)	0 (0%)	4 (2%)	1 (3%)	3 (9%)	
**Sexual behavior questions**								
**Days between last sex and interview (median, IQR)**	279	5 (3, 16)	6 (3,17)	5 (2, 16)	16 (6, 59)	14 (6, 36)	21 (6, 74)	<0.001
**Condom use at last sex**								
Yes	116	42 (20%)	14 (11%)	28 (36%)	74 (45%)	34 (37%)	40 (55%)	<0.001
No	259	169 (80%)	120 (89%)	49 (64%)	90 (55%)	57 (63%)	33 (45%)	
**>1 partner in past year**								
Yes	58	37 (18%)	23 (17%)	14 (18%)	21 (13%)	14 (15%)	7 (10%)	0.209
No	317	174 (82%)	111 (83%)	63 (82%)	143 (87%)	77 (85%)	66 (90%)	
**Sexual practices during pregnancy**								
Oral sex (F to M)	376	46 (22%)	35 (26%)	11 (14%)	40 (24%)	27 (30%)	13 (18%)	0.58
Oral sex (M to F)	376	38 (18%)	28 (21%)	10 (13%)	41 (25%)	27 (29%)	14 (20%)	0.11
Anal sex	375	12 (6%)	10 (8%)	2 (3%)	18 (11%)	12 (13%)	6 (8%)	0.06
Vaginal sex	376	206 (98%)	131 (98%)	75 (98%)	163 (99%)	90 (98%)	73 (100%)	0.41

### Differences between pregnancy and postpartum sex

**Fig 1:**
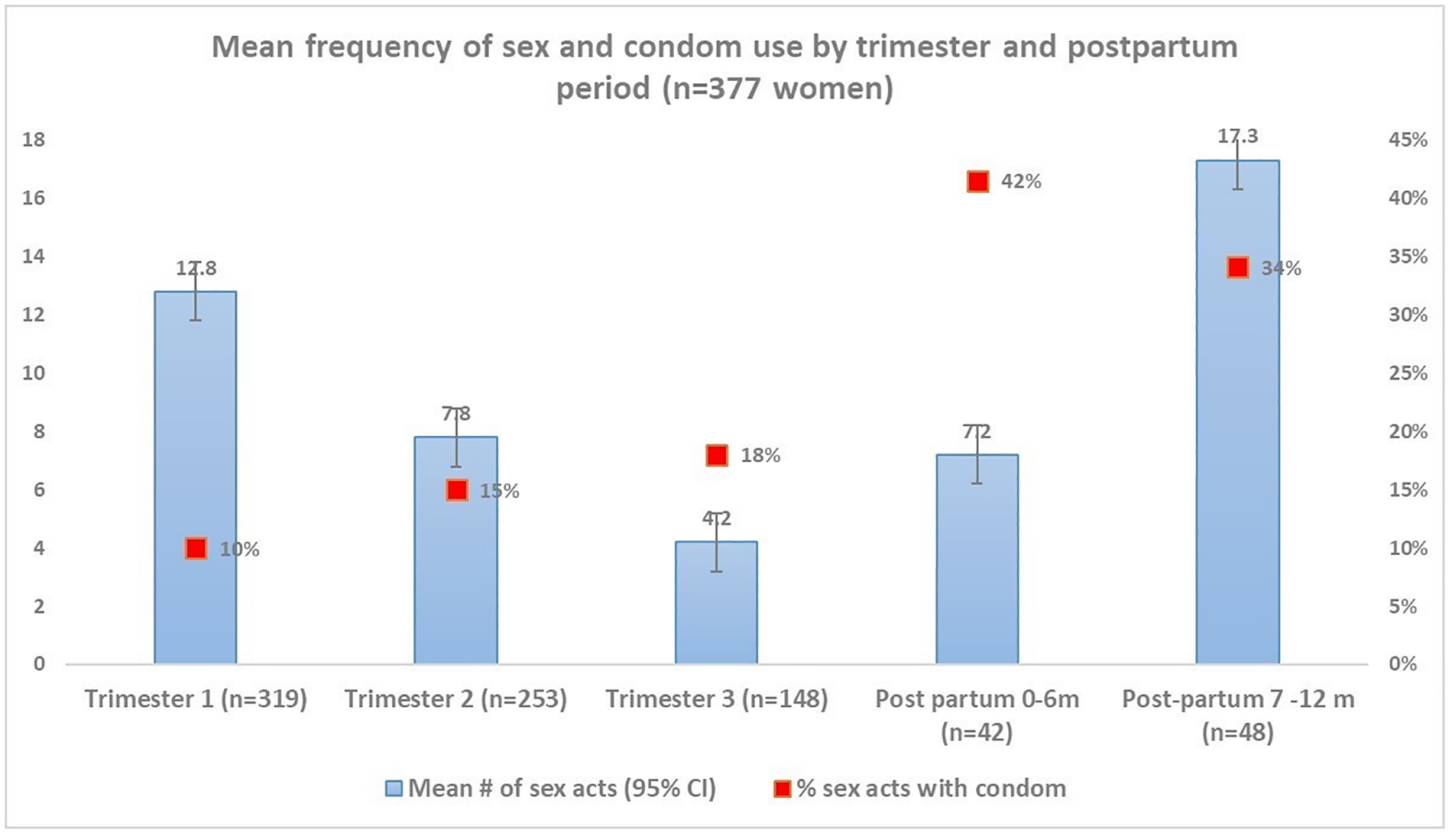
Mean frequency of vaginal sex acts per month by pregnancy vs. postpartum status and self-reported condom use (n = 377 women).

During pregnancy, 98% women reported vaginal sex (n = 369 of 376 women)), 23% female to male oral sex (n = 86), 21% male to female oral sex (n = 79), and 8% anal sex (n = 30). Though 98% of women reported vaginal sex during pregnancy, of the 165 postpartum women, 35% reported vaginal sex 0 to 6-months after birth (n = 58) and 88% of women reported sex 7 to 12-months after birth (n = 145). Most of the postpartum women reported having sex after giving birth, 8% reported resuming postpartum sex within 30-days of giving birth (n = 13), 10% reported resuming sex 1-2-months after birth (n = 17), 46% reported resuming sex 2-4-months after birth (n = 76), and 37% reported resuming sex later (≥5-months postpartum) (n = 61). About half of the postpartum women reported using a condom with first postpartum sex (71% of HIV-infected women [n = 20 of 28 women] vs. 37% among HIV-uninfected women [n = 10 of 27 women], p = 0.01). Overall, 15% of women reported having sex with another partner who was not the father of the index pregnancy/child in the past 12-months (n = 58), including 18% of pregnant women (n = 37) and 13% of postpartum women (n = 21). In all women who reported sexual activity (both pregnant and postpartum women), sex frequency per month varied by trimester with greatest mean sex acts occurring during first trimester (mean sex act = 12.8 in the first trimester vs. 8 in the second trimester and 4.2 in the third trimester) and increased again in postpartum period (mean sex acts = 7.2 from 0–6 months and 17 in 7–12 months) (Fig 1).

HIV-infected pregnant women were slightly older than HIV-uninfected women (median age = 30, IQR = 25, 33 vs. 27, IQR = 23, 32) and reported having no relationship with the father of their child more than HIV-uninfected pregnant or postpartum women (13% vs. 6% in pregnant women and 20% vs. 9% in postpartum women). HIV-infected pregnant women reported being employed 31% vs. 42% of HIV-uninfected women. There was no difference in alcohol use reporting by HIV status. Condom use at last sex was higher in HIV infected pregnant women (36%) and postpartum women (55%) compared with HIV-uninfected pregnant women (11%) and postpartum women (37%). HIV-infected women reported less anal sex in pregnancy (3%) and postpartum (8%) vs. HIV-uninfected pregnant (8%) and postpartum (13%) women. Approximately 21% of pregnant and postpartum women reported male-to-female oral sex Half of the pregnant and postpartum women interviewed reported sex within the past week before study enrollment and 69% reported condomless sex at last sex (20% among pregnant women vs. 45% of postpartum women, p<0.0001). Over 70% of pregnant or postpartum women who reported anal sex also reported condomless sex. More pregnant women reported having >1 sex partner compared to postpartum women (18% vs. 13% of postpartum women). Further, 36% of women suspected their partners of having other sex partners, and 24% were unsure whether their partner had other partners. Similarly, condom use varied by trimester, postpartum period and serostatus (Fig 1).

In multivariable models, pregnant women had increased odds of reporting condomless sex at last sex during pregnancy (aOR = 2.96; 95% CI = 1.84, 4.78) and ever having condomless sex during pregnancy/postpartum period (aOR = 2.65; 95% CI = 1.30, 5.44) compared to postpartum women, adjusting for age, HIV status and relationship with partner. Pregnant women had increased odds of reporting heavy alcohol use (≥5 drinks on more than one occasion) compared with postpartum women (aOR = 2.99, 95% CI = 1.39, 6.43) adjusted for age and HIV status (Table 2).

**Table 2:** Adjusted and unadjusted odds ratio for risk factors of HIV acquisition and transmission in pregnant and postpartum women.

	Unadjusted OR (95% CI)	Adjusted OR (95% CI)
**Model 1: Condomless sex at last sex**		
HIV-uninfected	3.21 (2.03, 508)	3.33 (2.04, 5.44)
Pregnant (ref: Postpartum)	3.23 (2.03, 5.12)	2.96 (1.84, 4.78)
Age (per year increase)	1.10 (0.98, 1.05)	1.03 (0.99, 1.08)
In relationship with father of child	1.28 (0.82, 2.02)	1.17 (0.92, 1.98)
Mother’s education high school or above(ref. below high school)	1.00 (0.64, 1.56)	1.03 (0.98, 1.36)
**Model 2: Ever reported condomless sex during pregnancy or postpartum period**		
HIV-uninfected	0.33 (0.21, 0.51)	0.30 (0.19, 0.48)
Pregnant (ref: Postpartum)	2.29 (1.86, 2.97)	2.65 (1.30, 5.44)
Age (per year increase)	1.01 (0.99, 1.06)	1.03 (0.99, 1.07)
**Model 3: Alcohol use during pregnancy (≥5 drinks on more than one occasion)**		
HIV-uninfected	1.25 (0.70, 2.24)	1.23 (0.91, 1.63)
Pregnant (ref: Postpartum)	2.90 (1.05, 7.50)	2.99 (1.39, 6.43)
Age (per year increase)	0.92 (0.87, 0.97)	0.98 (0.89, 1.03)

## Discussion

Our study identified behavioral risk for HIV transmission (to sex partners and infants) and acquisition during pregnancy and postpartum periods including heavy alcohol use, multiple partners, frequent condomless vaginal and anal sex with serodiscordant partners, and partners of unknown serostatus. Sexual risk behaviors changed significantly across pregnancy and postpartum periods. Reported sex frequency was highest during the first two trimesters of pregnancy and lowest for the third trimester and the first 6-months post-birth, but increased again after 6-months postpartum. Further, reported condomless sex was highest during pregnancy and lower during postpartum sex, which may reflect the desire to prevent pregnancy. Importantly, less than half of pregnant and postpartum women reported being married or cohabiting with the father of the index pregnancy/child, and 39% of women did not know their partner’s status and 15% reported more than one partner. Couple HIV testing and male partner HIV counseling and testing is important in the context of pregnant women because of the increased risk of HIV acquisition in pregnancy and risk of MTCT following seroconversion during pregnancy and breastfeeding periods. South Africa has several interventions to increase male testing and couples counseling and testing including self-testing and partner notification. Further, lack of marital or cohabiting relationship combined with multiple partners and lack of knowledge of partner’s serostatus have implications for the effectiveness of PMTCT programs, including regular HIV testing and condom promotion.

Our study demonstrated that among African pregnant and postpartum women sexual behaviors and risk changed significantly between trimester and between pregnancy and postpartum periods [4, 17–19]. Almost all the pregnant and postpartum women in our study reported sexual activity during pregnancy, but only 35% of women reported sex in the first 6-months post-birth. Our study found similar high-risk sex behaviors as found in other studies of pregnant and postpartum women in sub-Saharan Africa [4, 11, 18, 19]. Drake et al published a systematic review and meta-analysis of incident HIV during pregnancy and postpartum periods and the corresponding risk of vertical transmission which found that pregnancy and postpartum periods are times of persistent HIV risk which is like other “high-risk” cohorts [4]. Our study highlighted the same sexual risk behaviors that young women with multiple partners reported in South Africa. A recent study found that most young women reported inconsistent condom use both with their most recent main partner and casual sexual partners in the past 3-months, which is like our study, especially among pregnant women (80% reported condomless sex at last sex) [20]. Our data help to contextualize the behavioral risk surrounding high HIV incidence during pregnancy and postpartum periods as well as being one of the first studies to compare risk behaviors by stage of pregnancy and postpartum period.

Our study demonstrated that alcohol use in pregnancy was high (~30%) compared to other studies among pregnant African women. Further, 6% of women reported heavy or binge drinking, including consuming alcohol 6+ times in one occasion 2 or more times per week. Those results are important given the association of alcohol use and HIV acquisition in women [15]. One-fifth of pregnant and postpartum women reported male-to-female oral sex which may have implications for transmission of other pathogens. Pintye et al. developed a risk score for targeting pregnant and postpartum women for PrEP [21]. Our research on risk behaviors in pregnancy and postpartum periods, including alcohol use, condomless vaginal and anal sex, will help develop risk scores in high HIV incidence communities like South Africa.

For HIV-infected women, at-risk sex was reported in pregnancy and postpartum periods including multiple partners, condomless vaginal and anal sex, and heavy alcohol consumption. Only 56% of HIV-infected pregnant women were cohabiting or married, 13% were not in a relationship, and 18% reported having more than 1 sex partner in the past 12-months. Almost half of HIV-infected women did not know the serostatus of her partner and 18% were in serodiscordant relationships. Despite this lack of knowledge and serodiscordance, only 36% of women reported using a condom at last sex. Prior studies have demonstrated the risk of STIs and viremia during pregnancy on vertical HIV transmission [5–8]. There is an urgent need to develop interventions for HIV-infected women to: (1) achieve rapid viral suppression and (2) focus on preventing HIV transmission not only to infants but also to sex partners during and after pregnancy.

### Limitations

Our study was cross-sectional, so it did not allow us to study the same women across time. We used self-reported data that may be biased because of recall or respondent bias, and further research is needed to collect biomarkers to validate sex behavior and substance use among pregnant and breastfeeding women.

Despite these limitations, as efforts to eliminate vertical transmission intensify, it will become increasingly important to prevent primary HIV acquisition before (during peri-conception), during, and after pregnancy (during breastfeeding exposure). In addition, interventions are needed to prevent HIV transmission to sex partners during and after pregnancy. Interventions such as repeat HIV testing during antenatal and postnatal visits (including immunization visits), testing pregnant and breastfeeding women for acute HIV testing, testing male partners and couples testing [22], and condom promotion have had some limited impact on HIV incidence [23, 24]. However, the most effective way to eliminate vertical transmission is to prevent women from acquiring HIV. One strategy is to increase access and uptake of female controlled methods such as pre-exposure prophylaxis (PrEP) to peri-conception, pregnant, and breastfeeding HIV-uninfected women at risk of HIV acquisition. WHO recently released guidelines on PrEP for pregnant and breastfeeding women, stating that “PrEP can also be considered as an additional prevention choice for HIV-negative pregnant women who are at substantial risk of HIV infection, as part of a comprehensive prevention of mother to child transmission (PMTCT) package.” [25] Recent studies have demonstrated that PrEP would be a safe measure to use to reduce HIV acquisition and transmission during peri-conception, pregnancy and breastfeeding [26–28], and tools have been evaluated to allow for the identification of women who would most benefit from PrEP [21]. Despite WHO guidelines and high HIV incidence in pregnant South African women, PrEP remains contraindicated for pregnant women in South Africa. Data from studies like ours, which point to specific period of high risk and patterns of high-risk behaviors, are important for informing who and when should women get PrEP during pregnancy. Future research, including operation research, must evaluate how best to ensure access to this powerful prevention intervention in this vulnerable group [29]. Now is the time to evaluate how best to provide PrEP to vulnerable pregnant and breastfeeding women as there are few data on acceptability, initiation, and adherence in pregnant and breastfeeding women in Africa where the burden of HIV is greatest.

## Conclusion

Pregnant and breastfeeding women are at risk of HIV acquisition, and HIV acquisition currently significantly increases the probability of mother to child transmission of HIV. Our study identified that sex frequency, condomless sex, heavy alcohol use, and multiple partners varied across pregnancy and postpartum periods. Changing sexual risk presents previously unrecognized challenges to prevention efforts, including PrEP delivery. Access to female controlled interventions like PrEP, for which use may vary depending on sexual activity, is necessary.
